# CARING FOR PATIENTS IN ACUTE CARE LIVING WITH DEMENTIA: WHERE WE ARE AND WHERE WE NEED TO GO

**DOI:** 10.1093/geroni/igad104.0699

**Published:** 2023-12-21

**Authors:** Barbara Resnick

## Abstract

There are many challenges to caring for older adults living with dementia when hospitalized including medication management, pain identification and management, behavioral symptoms associated with dementia, and sedentary behavior. To help staff, particularly the nurses providing care to these patients, we developed Function Focused Care for Acute Care using the Evidence Integration Triangle (FFC-AC-EIT) to best address increasing physical activity, pain assessment and management, appropriate use of medications and management of behavioral symptoms associated with dementia. FFC-AC-EIT includes the implementation of four steps: (1) Environment and policy assessments; (2) Education of staff; (3) Establishing patient goals; and (4) Mentoring and motivating of staff, patients, and families. The testing of FFC-AC-EIT is ongoing as a cluster randomized controlled trial that to date has included 365 patients from 10 hospitals randomized to FFC-AC-EIT or FFC-AC-Education only. Eligibility of patients is based on being 55 years of age and older, admitted for a medical reason excluding COVID-19, and demonstrating evidence of dementia. This symposium will address the care and changes that occur during the hospital stay for these participants. Specifically, the following areas will be addressed: psychotropic medication use, participation in function focused care, the impact of physical activity on behavioral symptoms associated with dementia, and treatment of pain. The findings can be used to help guide researchers and clinicians in optimal ways to address the common challenges to care of hospitalized patients living with dementia. Presenters will provide information about future work needed to improve care across each of these areas.

